# Feasibility of Matrix-Matched Material for Determining Elements in Rice Flour by SN-ICP-MS and LA-ICP-MS

**DOI:** 10.3390/foods13111604

**Published:** 2024-05-22

**Authors:** Chunyapuk Kukusamude, Supalak Kongsri, Ratchadawan Tamklang, Sutthinun Taebunpakul

**Affiliations:** 1 Nuclear Technology Research and Development Center (NTRDC), Thailand Institute of Nuclear Technology (Public Organization), 9/9 Moo 7, Saimoon, Ongkharak, Nakhon Nayok 26120, Thailand; 2 Chemical Metrology and Biometry Department, National Institute of Metrology (Thailand), 3/4-5 Moo 3, Technothani, Klong Ha, Klong Luang, Pathum Thani 12120, Thailand

**Keywords:** rice, SN-ICP-MS, LA-ICP-MS, matrix-matched material, elemental analysis, calibration, pressed pellet

## Abstract

The preparation of matrix-matched material for elemental quantitative analysis in rice flour matrix is proposed here for the first time as part of a feasibility study using the SN-ICP-MS and LA-ICP-MS methods. It was prepared via the spiking process in colloidal solution of rice flour with different levels of arsenic (As), cadmium (Cd) and lead (Pb), followed by drying in a climatic chamber. Comparative studies of the results on external calibration and gravimetric standard addition ICP-MS approaches through the use of calibration standard solutions were discussed. Method bias from the external calibration method was investigated, demonstrating the systematic effect arising from the sample matrix. Characterizing the concentration of measurands was then reasonably proposed using the gravimetric standard addition ICP-MS. Using powdered rice matrix reference material for ICP-MS calibration following acid digestion, the study showed a good agreement of recovery studies. A feasibility study of the LA-ICP-MS method as a direct solid analysis performed on the matrix-matched standard was then discussed. In the study, large fluctuation of signals was found for constructing calibration curve, generating poor linearity, especially for As and Pb, although yttrium (Y) as internal standard was applied. This might be ascribed to a limited microscale of homogeneity, and particularly laser-induced preferential evaporation of volatile elements. Using a number of measured data points, the mean and median were statistically recommended to improve precision. An attempt to use of similar matrix in both standard and sample is a critical point to consider to minimize the elemental fractionation effect. The proposed approach to prepare matrix-matched material could be a potential means for achieving elemental quantitation.

## 1. Introduction

Rice is the main staple food especially consumed in most Asian countries [1]. Contaminants such as arsenic (As) and cadmium (Cd) are commonly known to be present in rice commodities. They are set in a regulation reliant on the Codex Alimentarius Commission [2]. To reduce the risk of consumption, monitoring the levels of contaminants is of paramount importance. Analytical methods that are accepted for analysts internationally, such as ISO and AOAC, have been applied, as they meet the performance criteria and requirements [3]. For example, atomic absorption spectrophotometry after dry ashing, based on AOAC 999.11, and inductively coupled plasma-mass spectrometry (ICP-MS), based on AOAC 2015.01, could be used for determining elements in food [4,5]. Nevertheless, based on improving measurement technology, the ICP-MS technique with microwave acid digestion has become of increasing interest, as it is capable of both multi-element detection and better quantitation. It is commonly accepted that testing laboratories mostly employ the external calibration method with calibration standard solutions for elemental analysis following acid digestion with samples. Since there is a difference between the calibration standard and sample nature, the matrix effect can be an issue that may influence the measurement results. Studies on slopes obtained from external calibration and standard addition may be necessary for checking whether there is any bias. APMP-APLAC joint proficiency testing (PT) programs for elemental analysis in food with metrological reference values were carried out for the assessment of the participants’ performance considering measurement uncertainties [6]. The metrological reference values traceable to SI were applied as PT assigned reference values, allowing for reliable evaluation of the participant performance. The evidence showing PT results regarding Cd and lead (Pb) in kimchi cabbage and wheat flour tended to have the negative bias compared to the reference values. This emphasizes the need for matrix-certified reference material or matrix-matched standards to be used for method validation as well as quality control.

Due to the fact that there is a difference arising from the nature of the sample matrix and calibration standard, matrix-matched standard may be an alternative for testing laboratories, at least for the purpose of checking method bias. However, there has been, up until now, a limited availability of commercial matrix-matched standards for cereal and cereal products. Very few publications suggest how to prepare and mimic real samples with different desired concentrations. A preparatory approach for matrix-matched material was then developed to investigate whether it was feasible for calibration or method validation in elemental determination by ICP-MS digestion method. When it functions as a calibration standard prior to use, rice flour matrix material with different levels needs to be well characterized in terms of accuracy (precision and trueness) with valid measurement method. It has not only been used for studies in solution nebulizer (SN)-ICP-MS; the prepared matrix-matched material was also investigated to see whether it could be used for solid-introduction purposes, particularly in laser ablation (LA)-ICP-MS analysis. Although direct X-ray analysis via micro-X-ray fluorescence (micro-XRF) and synchrotron-X-ray fluorescence (synchrotron-XRF), particle induced X-ray emission (PIXE) enables such determination [7,8,9], with access to instruments as a limitation. Therefore, LA-ICP-MS seems to be a more promising technique that is potentially capable of directly analyzing contaminants in ppb levels in solid surfaces, and it is known to improve atomization due to its lack of typical solution components of ICP (dry plasma). Another advantage of this technique is that it minimizes contamination or losses of analytes during the conventional digestion process [10]. With an acid-free analytical protocol, the technique appears to be environmentally friendly and time-effective for the quantification of selected elements of interest simultaneously [11,12,13,14,15,16]. However, the drawback of LA-ICP-MS analysis is the effect of non-recognized matrix interferences, particularly when standards of unmatched matrix are used, thereby influencing accuracy. This effect, called “elemental fractionation”, influences the abundances of the ions detected after *m*/*z* separation, which are occasionally not entirely representative of the composition of the original sample [17,18]. The lack of available matrix-matched CRM could hinder the accuracy and precision of this technique [19]. Therefore, this study aims to investigate the accuracy of LA-ICP-MS analysis under the preparation of matrix-matched material proposed for the determination of elements in rice flour.

In the literature review, there were different approaches found for preparing matrix-matched standards for LA-ICP-MS analysis, as follows. The calibration standard can be developed by synthesizing spiked agarose gels as the matrix-matched external standard and carbon as the internal standard (ISTD) for a plant-based and animal-based foods [20]. Another approach is to use available CRMs: unpolished rice flour CRM and NIES 10a, 10b, and 10c to make a pellet [1], with leaf CRMs, NIST1515, NIST1573a, and LGC-7162 [21]. The other approach is to prepare filter paper discs to serve as supports for the reference solutions [22]. An alternative approach was performed with the use of matrix-matched standard through matrix CRM with Teflon powder and pressing into a pellet for single-point calibration [23]. Rather than directly pressing the sample into a pellet, investigation of elemental imaging in a longitudinal section of a single rice grain was also carried out to classify the rice according to its origins and the types of samples [24].

To our knowledge, an approach to mimicking rice flour reference material in different levels of elements has not been published. Without the use of any support or binder, the application of in-house matrix-matched material prepared was therefore investigated to determine whether it fits for intended use in SN-ICP-MS and LA-ICP-MS analyses. As, Cd, and Pb are the toxic target elements that are probably found in rice as contaminants in certain levels. However, rhodium (Rh) and yttrium (Y) are rarely found in rice samples, and both were used for internal standard purposes. Rh was the first internal standard added and mixed with target elements to make different levels for calibration. This was intended to examine how the signal was directly proportional with the concentration increased in calibration. Whether there was any difference in individual element behavior was identified. Unlike Rh, Y was added as the second internal standard at a fixed concentration in all levels. It was designed to act as an actual internal standard to calculate the ratio obtained between analyte and internal standard signals.

## 2. Materials and Methods

### 2.1. Reagents, Standard Solutions, and Material

Ultrapure water (resistivity of 18.2 MΩ⋅cm), used for material cleaning and solution preparation, was purified using a Milli-Q water purification system (Millipore, Burlington, MA, USA). Nitric acid, Min. 67% (high purity for trace analysis), purchased from Reagecon (Shannon, Ireland) was used for sample digestion and solution preparation. For ICP-MS analysis, NIST standard reference material (SRM) solutions: SRM3103a, SRM3108, and SRM3128, traceable to SI units, functioned as calibration standard solutions for determining As, Cd, and Pb in rice flour, respectively. A mixture of ISTDs, including Ge, In, and Bi, was purchased from Agilent Technologies (Santa Monica, CA, USA). They were used to compensate for instrumental drift for the determination of As, Cd, and Pb, respectively, through ICP-MS analysis. Standard solutions NIST SRM 3144 (Rh), NIST SRM 3103a (As), SRM 3108 (Cd), and SRM 3128 (Pb) (NIST, Gaithersburge, MD, USA) were used for the preparation of calibration standards and matrix-matched material.

### 2.2. Preparation of Matrix-Matched Material

A white rice flour sample was obtained from a local supermarket (Bangkok, Thailand). A screening study using ICP-MS analysis proved that the sample contained insignificant levels of As, Cd, and Pb. To formulate a matrix-matched material, the spiking process was performed at different concentrations of target analytes, such as As, Cd, Pb, and Rh, together with Y as an ISTD in a rice flour colloidal solution, enabling the determination of calibration functions. Five levels of matrix-matched material were prepared, as shown in Table 1.

In the preparation of the matrix-matched material, to formulate different levels of standards, a mixture of standard solutions Rh, As, Cd, and Pb was added to the rice flour colloidal solution, as shown in Table 1. NIST SRM 3167a (Y) was subsequently added to the solution as an ISTD to compensate for the drift in LA-ICP-MS analysis.

For LA-ICP-MS analysis, a sample of 100 mg was pressed into a pellet without any binder under 10 t of pressure. The resultant pressed pellet (1 mm thick, 13 mm diameter) was then kept in a desiccator prior to analysis. The spatial distributions of analytes in the prepared pellets were not examined at this preliminary stage. Following characterization of the concentration of elements in material using gravimetric standard addition ICP-MS method, the prepared matrix-matched material was directly measured in 5 levels to construct a calibration curve through LA-ICP-MS analysis. Matrix CRMs as samples were then measured using a CPS signal against the calibration curve.

### 2.3. Instrumentation

The Thermo Scientific^TM^ iCAP TQ triple quadrupole ICP-MS (Thermo Fisher Scientific, Waltham, MA, USA), equipped with an ASX-560 autosampler, a MicroMist nebulizer, and a quartz cyclonic spray chamber fitted with a quartz injector tube was employed. The ICP-MS operating at an RF power of 1550 W was equipped with a nickel sample and skimmer cones. The flow rates of argon (99.999% purity), used as a plasma gas, nebulizer gas, and auxiliary gas, were 14 L min^−1^, 1.1 L min^−1^, and 0.8 L min^−1^, respectively. Oxide formation and doubly charged ions were monitored and kept below 2% and 3%, respectively. A MARS 6 microwave digestion system (CEM, Matthews, NC, USA) was used for acid digestion prior to ICP-MS analysis. For LA-ICP-MS analysis, the ICP-MS instrument was connected with a NWR213 Nd: YAG laser system (solid-state laser with frequency quadrupled to 213 nm, as it offers good performance and general use for most sample types (Elemental Scientific Lasers, Bozeman, MT, USA)). Argon (99.999% purity) and helium (99.999% purity) were used for plasma generation and to transport the ablated aerosol from the ablation cell to the ICP-MS, respectively. The NWR213 laser ablation system was coupled to an iCAP TQ ICP-MS. The iCAP TQ was configured with a high sensitivity interface to ensure the detection of analytes even in low concentrations and small amounts of ablated sample. Prior to the measurements, all plasma- and interface-related settings were tuned automatically and were fully tailored to LA-based sample introduction by using the autotune procedures provided. NIST 612 was used to optimize the sample introduction system and lens parameters for optimum sensitivity with oxide formation of ThO/Th lower than 1%. Important parameters influencing the ablation process and the analytical result, the spot size, fluence, and repetition, were also investigated.

### 2.4. Determination of the Proposed Matrix-Matched Material Using Microwave Digestion and SN-ICP-MS

For acid digestion, the rice flour sample was weighed and found to be approximately 0.5 g in a TFM vessel, in which 8 mL of nitric acid was added with the microwave digestion condition (temperature was ramped to 210 °C for 30 min and maintained for 30 min). The clear digestate obtained was then diluted with deionized water and accurately weighed to 50 g in a polypropylene tube. For the external calibration method, the calibration curve was carried out by measuring standards at different concentration levels: 0.5, 1, 5, 10, and 20 ng g^−1^ were obtained from the SRM standard solutions in 2% HNO_3_ with the use of online ISTD introduction via a mixing T piece. The results of CPS from the digested sample were compared against the calibration curve.

For the determination of As, oxygen (flow rate of 0.2163 mL/min) was used as a dynamic reaction gas to avoid the polyatomic interferences from ^40^Ar^35^Cl^+^ making a mass shift to *m*/*z* 91, while the analysis of Cd and Pb in standard mode was applied by monitoring at *m*/*z* 111, 112, and 114 for Cd; *m*/*z* 206, 207, and 208 for Pb; and *m*/*z* 73, 115, and 209 for Ge, In, and Bi, respectively. The Ge, In, and Bi elements functioned as ISTD for the analysis of As, Cd, and Pb, respectively.

For gravimetric standard addition, the proportion of elemental standard solution incrementally added to the digested sample was critically considered. The first point of standard solution was added to the sample solution in the curve, allowing elemental signals in the measured solution, typically in the range of 1.5–2 times greater than those in the measured solution, to appear before adding the standard. Three levels of elemental standard spiked with equal interval concentrations to the digested sample were employed for the ICP-MS measurement. Good linear regression (r^2^ ≥ 0.995 or better) was achieved for constructing a calibration curve to calculate the mass fraction of element in the sample, while ISTD, introduced online, was used for compensating the instrumental drift throughout the consecutive run of series.

The quality control measurements used were not limited to continuing calibration standard (CCS), but the calibration verification standard (CVS) was used with another source of calibration standard purchased from Agilent Technologies (Santa Monica, CA, USA), in which the acceptance criteria to check the instrumental drift were ±5% and ±10%, respectively. Matrix-certified reference materials which were also used as part of the method validation process were as follows: NIST SRM 1568b (rice flour), NMIJ CRM 7533-a (arsenic compounds and trace elements in brown rice flour), ΝΜΙJ CRM 7503-b (arsenic compounds and trace elements in white rice flour), and TRM-F-2003 (elements and arsenic species in white rice flour). 

### 2.5. LA-ICP-MS

Laser ablation is a promising technique that allows for direct solid analysis with a relatively small surface for sampling. There may be potential issues with sample inhomogeneity, so care must be taken with the sample material to compensate for any fluctuation. Thus, line and spot scanning were used as the sampling strategies to investigate. For LA-ICP-MS measurement, when spot scanning (85 µm diameter) was chosen, designing operation was performed in five clusters distributed on the sample’s surface. In each cluster, ten measurements of spots were carried out (5 clusters × 10 data points/cluster = 50 data points obtained). To use a large area, line scanning as sampling strategy can be chosen. The measurements were operated in five clusters distributed on the surface, in which five measurements (1 mm long) were conducted (5 clusters × 5 data points/cluster = 25 data points obtained). The mean of means or medians was appropriately used. For example, the mean of the sample means tended to be very close to the population mean. The variability in the sample means depended on the sample size, and it turns out that the variability in sampling means decreased with the number of clusters (n). When outlier results were achieved, median was a good strategy to utilize. The ICP-MS operation was adjusted following the manufacturer’s recommendations. The LA-ICP-MS operating conditions are presented in Table 2.

### 2.6. Sample Preparation

The preparation was performed by adding deionized water (50 mL) into rice flour (30 g) and stirring using a glass rod for at least 10 min. A mixture of standard solutions containing As, Cd, Pb and Rh with Y standard solution was spiked into colloidal solution to formulate the matrix-matched material, allowing continual stirring to ensure a good mix. The resultants were spread onto stainless racks (25 cm × 50 cm) and left to dry in a climatic chamber maintained at 60 °C and 30% relative humidity overnight. The dried samples were then pulverized using an agate mortar and placed into amber bottles. 

## 3. Results and Discussion

### 3.1. Elemental Determination and Characterization in Matrix-Matched Candidate Material

Matrix-matched standard enables accurate quantification without the need to correct bias errors caused by matrix effect. A simple preparation procedure of matrix-matched standards with the use of a powdered sample blank was proposed. It was prepared by spiking with elements of interest (As, Cd, Pb, and Rh), with different levels and ISTD (Y) chosen for the rice flour blank. This was intended to mimic sample and standard matching with the main sample for the standard preparation. This included homogenizing a sample blank material, spiking it with target elements, then blending, drying, pulverizing, and bottling it in a bottle.

*In-house* matrix-matched material was then developed here for the first time to investigate the possibility to utilize it to develop the measurement capability of testing laboratories for calibration, method validation, and quality control purposes. This was initially performed with external calibration and gravimetric standard addition ICP-MS measurement (shown in Table 3). The results of those calibrations were then compared. The results of the external calibration method indicated that it was affected by the matrix effect, showing a negative bias, particularly for As and Cd measurands. Nevertheless, it seemed to be insignificantly affected for Pb analysis. One of the common strategies for external calibration to minimize the matrix effect is to conduct further dilution to obtain more accurate measurements. Additionally, standard addition is commonly known to compensate for the matrix effect. The slope of the standard addition was lower than that of the external calibration method, suggesting a suppression effect arising from the sample matrix.

The results of method recoveries were investigated using matrix CRM: SRM 1568b (brown rice), NMIJ CRM 7533-a (brown rice), and NMIJ CRM 7503-b (white rice). The recovery obtained from standard addition provided more reliable results than external calibration for ICP-MS analysis. However, recovery studies for Pb analysis were not evaluated, as there were no certified values given in the certificates. The repeatability of the matrix-matched material in each level was in the ranges of 1.1–1.5%, 0.7–4.5%, and 1.1–4.5% for As, Cd, and Pb, respectively (*n* = 3). Instrumental (i) LOQs were calculated based on average (blank) + 10 × So (blank). This was carried out by measuring blank samples with the analyte at the estimated LOQ and calculating the average and standard deviation of the results. The iLOQs for As, Cd, and Pb were tested in terms of accuracy. The acceptance criteria include a recovery range of 80–110% and precision not exceeding 10%. Method LOQs were calculated based on the dilution factor (100 times) multiplied with iLOQ, which were 0.0064, 0.0064, and 0.0034 mg/kg for As, Cd, and Pb, respectively.

The protocol used for determining the moisture content is described as follows: A minimum of three separate portions (recommended size to be about 0.5 g each) of the sample were taken and dried at 95 °C for 12–16 h, then balanced in a desiccator to room temperature. Dry mass correction was carried out at the same time as the test sample portions were analyzed. The difference in the mass before and after drying was the assumed moisture content. Matrix-matched material determined the moisture content at approximately 8%. The results obtained from gravimetric standard addition ICP-MS, as matrix compensation considered, were therefore used to obtain the characterization values of As, Cd, and Pb in this stage.

### 3.2. ICP-MS Results Obtained from Matrix-Matched Material as Calibration

Following characterization of the concentration through gravimetric standard addition ICP-MS, matrix-matched material was then digested and made up with ultrapure water to obtain a dilution factor similar to the matrix CRM as a sample (0.5 g digested to 50 g diluted). In this study, they functioned as matrix-matched standards. A calibration curve was then constructed using the obtained results. The Y axis was obtained from the following equation $\frac{Analyte\;signal}{ISTD\;signal}$ × $\frac{Final\;digest\;(g)}{Sample\;digest\;(g)}$ × dry mass correction, whereas the X axis was obtained from the characterized concentration in matrix-matched material on a dry-mass basis (µg kg^−1^).

**Figure 1:**
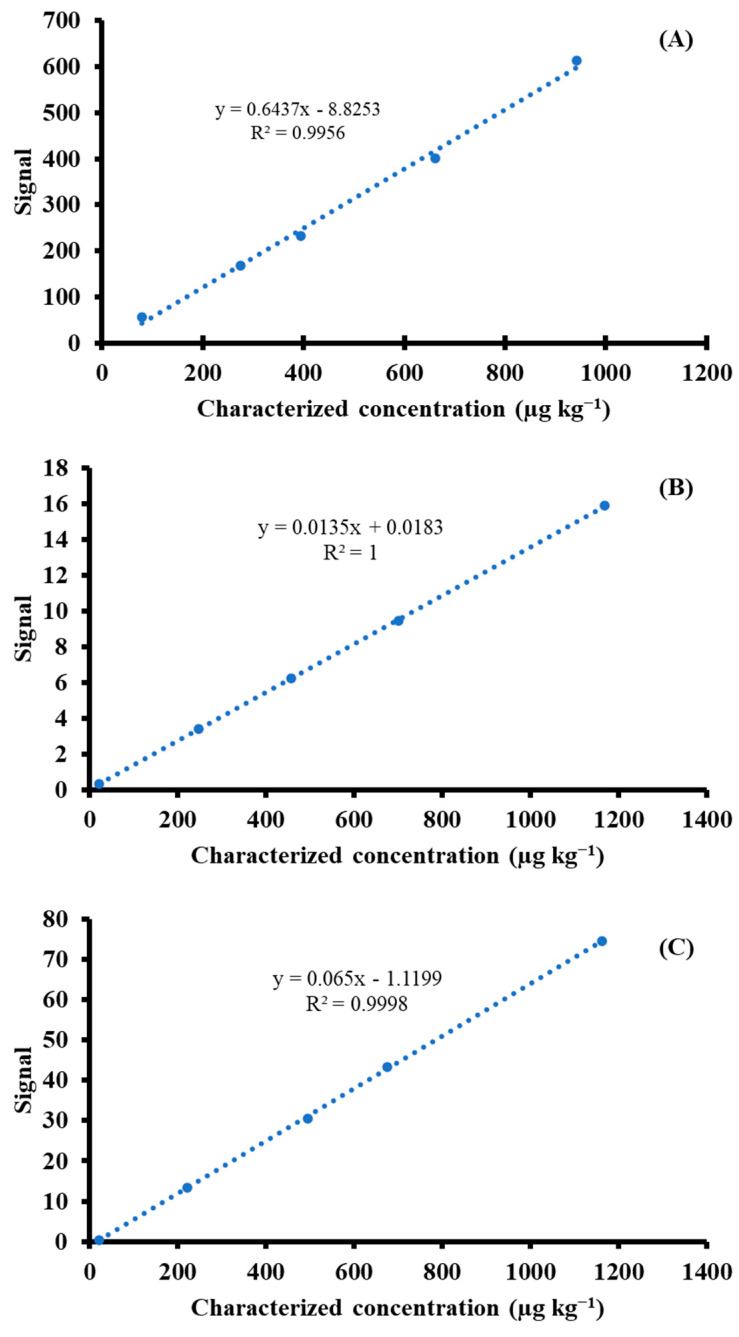
Calibration plots for (**A**) As, (**B**) Cd, and (**C**) Pb determination using matrix-matched material. The y and x represent (analyte/ISTD) Ratio × (Final digest (g)/sample digest (g)) × dry mass correction and the characterized concentration in matrix-matched material on a dry−mass basis (µg kg^−1^), respectively.

The measured results of CRMs were calculated as the linear equations of calibration curves (as shown in Figure 1) with a coefficient of determination greater than 0.995, showing good agreement with the characterized values, in which acceptable recovery was in the range of 80–110% [25], as depicted in Table 4. The obtained recoveries for As and Cd were in the ranges of 96–101% and 97–101%, respectively. The study suggested that systematic effects could be minimized using the matrix-matched material proposed to function as calibration. This experiment showed an alternative method of potentially applying matrix-matched material to construct external calibration rather than the routine use of standard solution. This method can provide insignificant differences in the results compared to gravimetric standard addition ICP-MS by SRM calibration solution; however, this occurs at the expense of higher uncertainty derived from matrix-matched standard and extra digestion time.

The results indicate that the preparatory method of matrix-matched material development is a potential means for preparing samples to use not only for calibration, but also for PT material or QC checks. Regarding the preparation of matrix CRMs and matrix-matched standard, in compliance with international standard ISO 17034: 2016 [26], it principally further requires a metrologically valid procedure prior to use for measurement. Thus, the procedure for using matrix-matched material for external calibration for the determination of As, Cd, and Pb was proven to enable accurate quantification without the need to correct bias errors caused by matrix effects.

To reduce the digestion time and avoid contamination, the use of LA-ICP-MS with in-house matrix-matched material could be worthwhile to further investigate possibly for the use for direct, solid introduction of ICP-MS measurement.

### 3.3. LA-ICP-MS Results Obtained from Matrix-Matched Calibration

A challenge in LA-ICP-MS analysis is known as the “fractionation effect”. This effect is supposed to be minimized by the use of a matrix-matched standard similar to the sample’s composition, which is largely reliant on the sample matrix type. Preparation of such standards is, therefore, a challenging process in LA-ICP-MS for quantitative analysis, as the calibration is a key factor in its capabilities. In attempt to minimize the elemental fractionation effect, an approach to solve the problem by mimicking matrix matching was investigated. The powdered matrix candidate material used for calibration standards, was therefore based on the spiked rice flour considered in the study. 

Although the use of a matrix-matched standard for LA-ICP-MS analysis may have been derived from matrix CRM availability, limited levels of calibration standard were obtained. The approach to prepare a matrix-matched sample without any binder, as described earlier, was then proposed. The main sample components were used as a base for the standard preparation. As mentioned earlier regarding the preparation of the matrix-matched material, it was believed that elements in rice flour would be more homogenized than in the original sample. 

**Figure 2:**
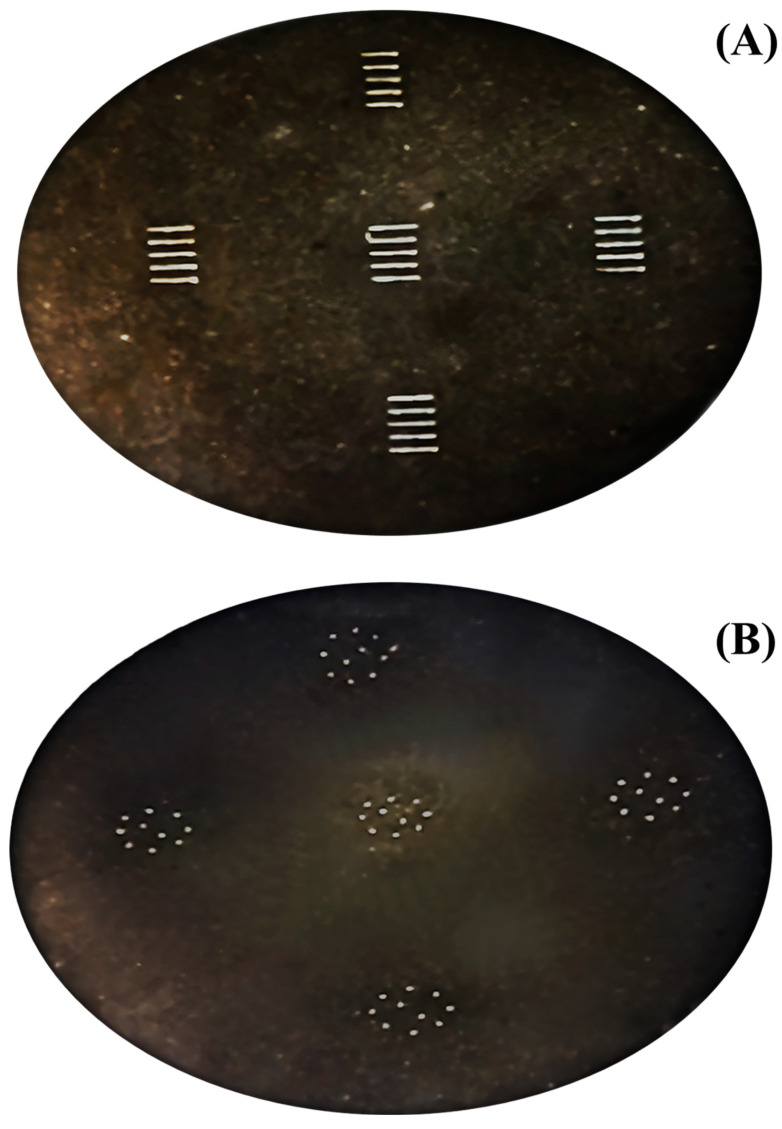
Photographs of pressed pellets of matrix-matched material after laser ablation in (**A**) 5 clusters of 5 lines (1 mm long) and (**B**) 5 clusters of 10 spots (85 µm).

**Figure 3:**
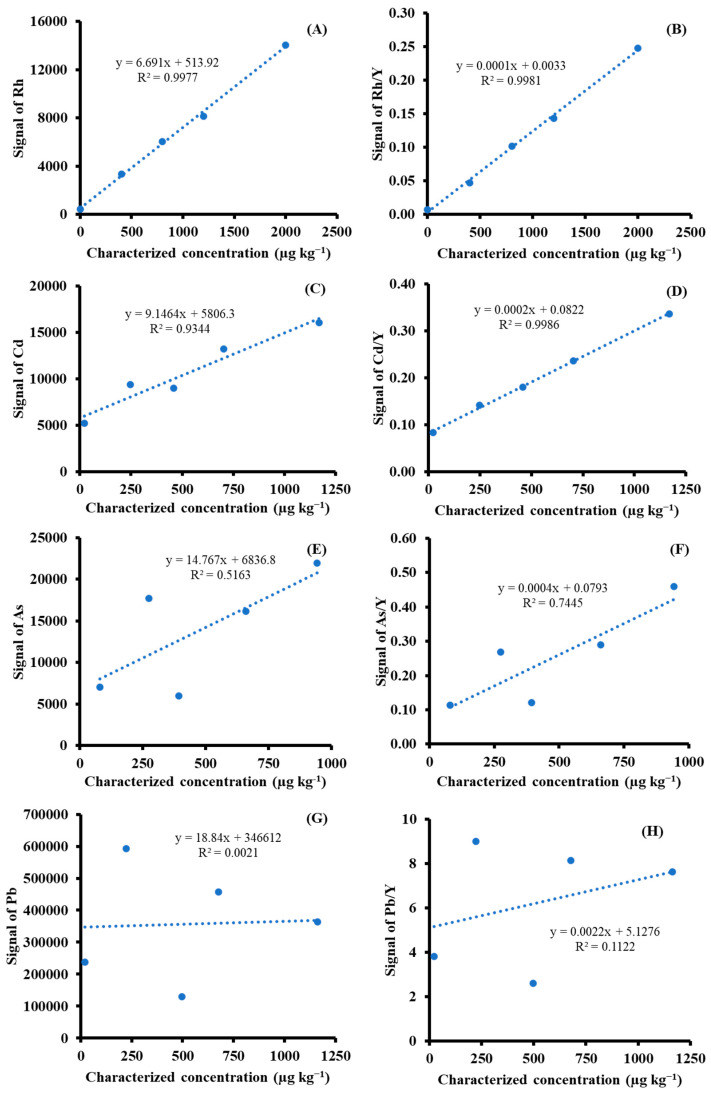
Calibration curves for (**A**,**B**) Rh, (**C**,**D**) Cd, (**E**,**F**) As, and (**G**,**H**) Pb of matrix-matched material for LA−ICP−MS (**A**,**C**,**E**,**G**), without and (**B**,**D**,**F**,**H**) with the use of Y as ISTD. Without ISTD−calibration curves were constructed by CPS of analyte signals against characterized concentration in matrix-matched material on a dry-mass basis (µg kg^−1^). With ISTD−calibration curves were constructed according to the ratio (analyte/ISTD signals) against characterized concentration in matrix−matched material on a dry-mass basis (µg kg^−1^).

The precision and accuracy of LA-ICP-MS are worse than those of conventional SN-ICP-MS. As the results from LA-ICP-MS are known to cause large variations, to improve precision, strategies for quantification by LA-ICP-MS were proposed. The use of line and spot scans that were placed and distributed in a pressed sample (appeared in Figure 2) are comparatively discussed. An additional approach was to use ISTD Y, which is fortified during the matrix-matched preparation. ISTD is used in order to improve precision. Experimentally, in each standard or sample, five clusters, in which ten spots or five lines are present, were analyzed. Evaluation results using the statistical approach, including the mean of means and median of medians for the clusters, were used to represent the measured values in each matrix-matched material. The use of the median was encouraged to avoid the effects of outliers. Using the aforementioned strategies made it possible to construct calibration curves with good linearity for Rh and Cd; however, this was not sufficient for As and Pb, as depicted in Figure 3. The graphical plot results for the elements under investigation were based on spot-scanning measurements. The use of Y as the ISTD improved the linearity of calibration curves of Rh, Cd, As, and Pb with coefficients of determination (r^2^) of 0.9981, 0.9986, 0.7445, and 0.1122, respectively. Poor correlations of the calibration curves were observed for Pb and As. This might be ascribed to the laser-induced preferential evaporation of Pb, which is higher than those of As, Cd, and Rh, respectively. 

#### 3.3.1. Internal Standardization

The application of internal standardization was proposed to improve precision. Internal standardization was used to correct multiplicative effects (sensitivity drift, differences of mass ablated, and some matrix effects). Signals for quantitative analysis were normalized using an appropriate ISTD (Y). This compensates for the signal variation that occurs during the interaction of the laser beam with the sample in the ablation cell, as well as the transportation of ablated material and reactions in the ICP plasma. ISTD is then crucial for precision and trueness of determination. Although carbon is one of the elements most often used for internal standardization during the analyses of biological samples, as it is considered the most abundant element in rice samples, it may not be a good candidate for an ISTD because its behavior is different than other measurands [1]. 

The measurands in this study, such as As, Cd, and Pb, are supposed to have different diffusion loss of gaseous particulate species when compared to carbon [27]. Carbon is therefore considered unsuitable for ISTD, as it is not related in stability with the measurand signals [18]. Although laborious, the addition of ISTD into the standard and sample material was carried out to correct such effects. Ideally, it is naturally absent in the sample, or chosen at a trace level. ^89^Y, an ISTD of mid-range masses that is more similar to other analytes in terms of ionization potential, may be a choice. However, the study suggests that its stability appears not to be related to As and Pb due to behavior differences in the degree of volatilization.

#### 3.3.2. Spot/Line Scanning and Mean/Median Evaluation

**Figure 4:**
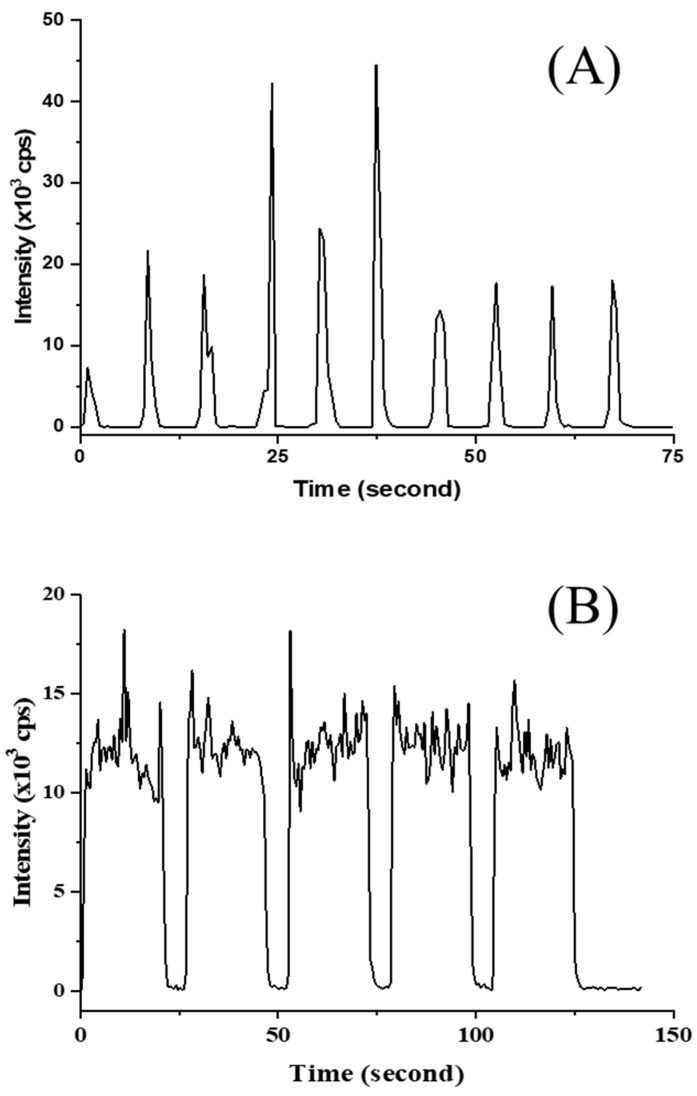
Signal plots of ^112^Cd intensities measured in matrix-matched material during LA-ICP-MS for (**A**) spot scanning (85 µm) in one cluster (10 spots) and (**B**) line scanning (1 mm long) in one cluster (5 lines).

Measurement of Cd on spot- and line-scanning showed the profiles given in Figure 4. A calibration curve, performed in no-gas mode ranging from 20 µg kg^−1^–1000 µg kg^−1^, was constructed with the help of Y as an ISTD to compensate for instrumental drift and the matrix effect. Although the calibration of Cd appeared to be of adequate linearity, the study showed sufficient recovery for a similar sample matrix (See Table 5). However, the Cd results did not appear in agreement with other matrix CRMs, such as NIST SRM 1568b, NMIJ CRM 7533-a, and NMIJ CRM 7503-b (results not presented). High variation of LA-ICP-MS was occasionally observed, although a number of measurements were performed. Practically, matrix-matched material appeared to offer good results for liquid-sample introduction by ICP-MS rather than solid-sample introduction by LA-ICP-MS. In the case of LA-ICP-MS measurement, a greater number of measurements are strongly recommended for Cd analysis, with the knowledge that the prepared matrix-matched material may not be applicable in a diverse of rice matrices. The reason is that solid standards are inhomogeneous on the microscale to some extent, generating poor precision. Major unexpected results could be explained by the elemental fractionation effect.

#### 3.3.3. Explanation

The unexpected results might be ascribed to elemental fractionation and matrix effects that may occur simultaneously, leading to LA-ICP-MS signals that are not representative of the elemental composition of the sample investigated. The study supports the review [10,18] of challenges due to the elemental fractionation effect, which is believed to be a very complex process. This takes place during aerosol formation in the ablation chamber, as well as transport of the aerosol into the ICP during all reactions in the ICP plasma. These effects are mainly sample matrix-dependent, and this suggests that the standards used for calibration should accurately match the sample matrix. Some non-stoichiometric effects influencing sample masses are therefore defined as fractionation. This includes laser-induced preferential evaporation of volatile elements, particle size-dependent elemental differentiation, and isotopic fractionation. The sensitivity or absolute signal intensity can vary significantly for samples with the same analyte concentration, but different matrix compositions and/or physical properties. The pellet standards must be prepared specifically for each sample matrix [10,18]. The ideal conditions for analyzing solid samples using LA-ICP-MS, as suggested in the review, could be as follows [7]: (i) The stoichiometric composition of ablated aerosol should be the same as that in the solid sample. (ii) Only particles of uniform sizes should be formed during the ablation process and transported without losses to the ICP torch. (iii) The particles should be small enough to be completely atomized in plasma without any fractionation effect. One of the experimental works performed using particle nebulization ICP-MS method showed interesting findings. Although it was principally different from transportation efficiency by laser ablation, it was found that very fine particles (~1 µm) can be achieved to ensure efficient particle transportation, ionization, and recoveries comparable with those of the standard solutions containing equivalent concentrations of the analyte [28]. This emphasizes that sufficient small particles are obtained with and uniform sizes during the process. This study can be used to support the elemental fractionation effect derived from the differences in behavior during ablation of powdered samples from mimicking material.

## 4. Conclusions

The preparation method for matrix-matched standard of rice flour will be beneficial for the future of CRM development. It can be prepared in measurand levels, as intended in different types of powdered matrix, and is initially prepared from rice flour colloidal form, followed by spiking different levels from a mixture standard solution. Unlike routine use of standard solution, the matrix-matched material after acid digestion was demonstrated to function as a calibration standard for SN-ICP-MS analysis. The advantage of this method is that, when media of both standards and samples are in close similarity, the matrix effect can be effectively compensated for. The measured results of matrix CRMs using the external calibration ICP-MS method showed very good agreement with their certified values. Due to the presence of the matrix effect, the use of a standard solution for calibration exhibited a 20% negative bias if dilution was insufficient, especially for As and Cd measurements. For LA-ICP-MS measurement, the development of LA-ICP-MS was found to be hampered by microscopic homogeneity. The study suggests that the measurement suffered from large fluctuation, requiring a greater number of measurements. Strategies such as ISTD (Y) and statistical selection (mean of means and medians) need to be taken into consideration. The measurement results showed the potential for the analysis of some elements, such as Rh and Cd, which may share some properties in common, whereas certain elements such as As and Pb were found to be challenging to cope with, as poor calibration curves were observed. Although calibration for Cd provided sufficient linearity, the LA-ICP-MS-based analysis was found to be specifically dependent on the rice sample. The matrix of the standard should ideally match that of the sample. There has been great concern about the difference in ablation behavior between the sample and standard as a result of the large variety of rice samples. There have still been specific issues with LA-ICP-MS that need further investigation and improvements, especially calibration procedures. To gain more benefits when using the developed matrix-matched material, independent techniques such as X-ray fluorescence (XRF) and particle-induced X-ray emission (PIXE) may be employed. 

## Figures and Tables

**Table 1:** Preparing composition for matrix-matched material.

Composition	Amount (g or mL)				
	Matrix-Matched Material Level 1	Matrix-Matched Material Level 2	Matrix-Matched Material Level 3	Matrix-Matched Material Level 4	Matrix-Matched Material Level 5
Rice flour	30.0 g	30.0 g	30.0 g	30.0 g	30.0 g
Deionized water	50 mL	50 mL	50 mL	50 mL	50 mL
Mixture of standard solutions (6 mg kg^−1^ of As, Cd, and Pb and 12 mg kg^−1^ of Rh)	0 mL equivalent to 0 mg kg^−1^ for As, Cd and Pb and 0 mg kg^−1^ for Rh	1 mL equivalent to 0.2 mg kg^−1^ for As, Cd and Pb and 0.4 mg kg^−1^ for Rh	2 mL equivalent to 0.4 mg kg^−1^ for As, Cd and Pb and 0.8 mg kg^−1^ for Rh	3 mL equivalent to 0.6 mg kg^−1^ for As, Cd and Pb and 1.2 mg kg^−1^ for Rh	5 mL equivalent to 1.0 mg kg^−1^ for As, Cd and Pb and 2.0 mg kg^−1^ for Rh
Y standard solution (12 mg kg^−1^ of Y as ISTD)	1 mL equivalent to 0.4 mg kg^−1^ Y	1 mL equivalent to 0.4 mg kg^−1^ Y	1 mL equivalent to 0.4 mg kg^−1^ Y	1 mL equivalent to 0.4 mg kg^−1^ Y	1 mL equivalent to 0.4 mg kg^−1^ Y

**Table 2:** LA-ICP-MS operating conditions.

ICP-MS (iCAP TQ)	
RF power (W)	1550
Plasma gas (L min^−1^)	14
Auxiliary gas (L min^−1^)	0.8
Nebulizer gas (L min^−1^)	0.8
Signal-collecting time per cluster (s)	80 (spots), 140 (lines)
**LA system (NWR213)**	
Wavelength (nm)	213
Energy (J/cm^2^)	5.82
Repetition rate (Hz)	10
Crater diameter (µm)	85
Ablation mode	Spot, line
Focus	Sample surface
Pre-ablation	Off
Carrier gas, He (L min^−1^)	0.8
Total time per cluster (s)	111 (spots), 145 (lines)

**Table 3:** Comparative result of As, Cd, and Pb measured by external calibration and gravimetric standard addition ICP-MS methods.

Sample	As Results (*n* = 3) (µg kg^−1^)		Cd Results (*n* = 3) (µg kg^−1^)		Pb Results (*n* = 3) (µg kg^−1^)	
	External Calibration ICP-MS (±SD, *n* = 3)	Gravimetric Standard Addition ICP-MS (±SD, *n* = 3)	External Calibration ICP-MS (±SD, *n* = 3)	Gravimetric Standard Addition ICP-MS (±SD, *n* = 3)	External Calibration ICP-MS (±SD, *n* = 3)	Gravimetric Standard Addition ICP-MS (±SD, *n* = 3)
Matrix-matched material Level 1	65 ± 1	79 ± 1	19 ± 1	22 ± 1	21 ± 1	22 ± 1
Matrix-matched material Level 2	228 ± 3	274 ± 4	206 ± 1	247 ± 1	212 ± 3	223 ± 3
Matrix-matched material Level 3	328 ± 4	394 ± 5	382 ± 3	459 ± 3	470 ± 6	496 ± 6
Matrix-matched material Level 4	551 ± 7	661 ± 8	585 ± 5	702 ± 6	641 ± 8	677 ± 8
Matrix-matched material Level 5	786 ± 9	943 ± 10	974 ± 9	1169 ± 11	1103 ± 12	1163 ± 13
SRM 1568b (brown rice flour)	238 ± 4 (83.5%)	285 ± 5 (100%)	18 ± 1 (80.4%)	22 ± 1 (98.2%)	26 ± 1	27 ± 1
- Certified value	285 ± 14		22.4 ± 1.3		Not certified	
NMIJ CRM 7533-a (brown rice flour)	517 ± 8 (82.1%)	621 ± 9 (98.6%)	229 ± 2 (83.9%)	275 ± 2 (101%)	25 ± 1	26 ± 1
- Certified value	630 ± 20		273 ± 7		Not certified	
NMIJ CRM 7503-b (white rice flour)	126 ± 2 (76.8%)	152 ± 3 (92.7%)	333 ± 1 (74.3%)	434 ± 1 (96.9%)	21 ± 1	23 ± 1
- Certified value	164 ± 5		448 ± 16		Not certified	

**Table 4:** Results of CRMs with ICP-MS using matrix-matched material for calibration.

Matrix Certified Reference Material	Results Obtained from ICP-MS (µg kg^−1^)		
	As, Recovery	Cd, Recovery	Pb, Recovery
SRM 1568b (brown rice flour)	289 ± 6, 101%	22 ± 1, 97%	30 ± 1, N/A *
NMIJ CRM 7533-a (brown rice flour)	605 ± 31, 96%	276 ± 1, 101%	29 ± 2, N/A *
NMIJ CRM 7503-b (white rice flour)	164 ± 4, 100%	435 ± 1, 97%	26 ± 4, N/A *

* N/A: Not applicable. Since Pb is not certified in listed CRMs, recovery cannot be calculated.

**Table 5:** Results of CRMs with LA-ICP-MS with the use of matrix-matched material for calibration.

Sample	Cd Results of LA-ICP-MS (*n* = 3), % Recovery
ΤRM-F-2003 (white rice flour)	22 ± 5, 118%

## Data Availability

The original contributions presented in the study are included in the article, further inquiries can be directed to the corresponding authors.

## References

[1] Baba K., Watanabe E., Eun H., Ishizaka M. (2003). Direct determination of cadmium in rice flour by laser ablation-ICP-MS. J. Anal. At. Spectrom..

[2] *CXS 193-1995*; Codex Alimentarius, General Standard for Contaminants and Toxins in Food and Feed. Adopted in 1995. Revised in 1997, 2006, 2008, 2009. Amended in 2010, 2012, 2013, 2014, 2015, 2016, 2017, 2018, 2019, 2021, 2022, 2023. https://www.fao.org/fao-who-codexalimentarius/sh-proxy/en/?lnk=1&url=https%253A%252F%252Fworkspace.fao.org%252Fsites%252Fcodex%252FStandards%252FCXS%2B193-1995%252FCXS_193e.pdf.

[3] (1999). Codex Alimentarius, Recommended Methods of Analysis and Sampling. Adopted in 1999. Amended 2015.

[4] (2006). Lead, Cadmium, Copper, Iron, and Zinc in Foods. Atomic Absorption Spectrophotometry after Dry Ashing.

[5] (2015). Heavy Metals in Food. Inductively Coupled Plasma-Mass Spectrometry, First Action 2015.

[6] Kim S.H., Cho H., Heo S.W., Hwang E. (2023). APMP-APLAC joint proficiency testing programs for elemental analysis in food with metrological reference values: Assessment of participants’ performance considering measurement uncertainties. Talanta.

[7] Meharg A.A., Lombi E., Williams P.N., Scheckel K.G., Feldmann J., Raab A., Zhu Y., Islam R. (2008). Speciation and localization of arsenic in white and brown rice grains. Environ. Sci. Technol..

[8] Lombi E., Scheckel K.G., Pallon J., Carey A.M., Zhu Y.G., Meharg A.A. (2009). Speciation and distribution of arsenic and localization of nutrients in rice grains. New Phytol..

[9] Carey A.M., Lombi E., Donner E., de Jonge M.D., Punshon T., Jackson B.P., Guerinot M.L., Price A.H., Meharg A.A. (2012). A review of recent developments in the speciation and location of arsenic and selenium in rice grain. Anal. Bioanal. Chem..

[10] Miliszkiewicz N., Walas S., Tobiasz A. (2015). Current approaches to calibration of LA-ICP-MS analysis. J. Anal. At. Spectrom..

[11] Sussulini A., Becker J.S., Becker J.S. (2015). Laser ablation ICP-MS: Application in biomedical research. Mass Spectrom. Rev..

[12] Voss M., Nunes M.A.G., Corazza G., Flores E.M.M., Müller E.I., Dressler V.L. (2017). A new approach to calibration and determination of selected trace elements in food contact polymers by LA-ICP-MS. Talanta.

[13] Wagner B., Syta O., Kepa L., Bulska E., Segal I., Halicz L. (2018). Evaluation of the role of matrix matching for LA-ICP-MS calibration approaches in quantitative elemental analysis of tooth enamel. J. Mex. Chem. Soc..

[14] Jiang L., Zhou J., Guo W., Jin L., Hu S. (2023). Multi-element analysis of solid food materials via mixed standards pellet laser ablation inductively coupled plasma mass spectrometry. J. Food Compos. Anal..

[15] Cui Z., He M., Chen B., Hu B. (2023). In-situ elemental quantitative imaging in plant leaves by LA-ICP-MS with matrix-matching external calibration. Anal. Chim. Acta.

[16] Zhou J., Guo W., Hu Z., Jin L., Hu S. (2024). Evaluation of an internal standard-free laser ablation-ICP-OES method for elemental analysis in solid food samples. J. Food Compos. Anal..

[17] Longerich H.P., Günther D., Jackson S.E. (1996). Elemental fractionation in laser ablation inductively coupled plasma mass spectrometry. Fresenius’ J. Anal. Chem..

[18] Limbeck A., Galler P., Bonta M., Bauer G., Nischkauer W., Vanhaecke F. (2015). Recent advances in quantitative LA-ICP-MS analysis: Challenges and solutions in the life sciences and environmental chemistry. Anal. Bioanal. Chem..

[19] Günther D., Hattendorf B. (2005). Solid sample analysis using laser ablation inductively coupled plasma mass spectrometry. TrAC Trends Anal. Chem..

[20] Li Y., Guo W., Hu Z., Jin L., Hu S., Guo Q. (2019). Method Development for Direct Multielement Quantification by LA-ICP-MS in Food Samples. J. Agric. Food Chem..

[21] Papaslioti E.M., Parviainen A., Román Alpiste M.J., Marchesi C., Garrido C.J. (2019). Quantification of potentially toxic elements in food material by laser ablation-inductively coupled plasma-mass spectrometry (LA-ICP-MS) via pressed pellets. Food Chem..

[22] Pereira C.K., Neves V.M., Hidrich G.M., Faccin H., Pozebon D., Dressler V.L. (2023). Imaging of Elements Distribution in Rice by Laser Ablation Inductively Coupled Plasma Mass Spectrometry. Braz. J. Anal. Chem..

[23] Tibi M., Heumann K.G. (2003). Isotope dilution mass spectrometry as a calibration method for the analysis of trace elements in powder samples by LA-ICP-MS. J. Anal. At. Spectrom..

[24] Promchan J., Günther D., Siripinyanond A., Shiowatana J. (2016). Elemental imaging and classifying rice grains by using laser ablation inductively coupled plasma mass spectrometry and linear discriminant analysis. J. Cereal Sci..

[25] AOAC International (2016). Guidelines for Standard Method Performance Requirements AOAC Official Methods of Analysis.

[26] (2016). General Requirements for the Competence of Reference Material Producers.

[27] Todoli J.-L., Mermet J.M. (1998). Study of polymer ablation products obtained by ultraviolet laser ablation-inductively coupled plasma atomic emission spectrometry. Spectrochim. Acta Part B At. Spectrosc..

[28] Shi C., Guo W., Jin L., Hu S. (2020). Analysis of rice and wheat flour by particle nebulization ICP-MS. RSC Adv..

